# Associations Among Digital Health Literacy, Lifestyle Factors, and Cardiovascular Health in Black and Hispanic Communities: Cross-Sectional Study

**DOI:** 10.2196/60654

**Published:** 2025-12-08

**Authors:** Joyline Chepkorir, Wuraola Olawole, Hailey Miller, Nana Ofori Adomako, Elizabeth Louise Andrade, Gloria Cain, Diana-Lyn Baptiste, Oluwabunmi Ogungbe, Daniel Mullins, Roger Clark, India Kutcherman, Cheryl R Himmelfarb

**Affiliations:** 1 School of Nursing, Johns Hopkins University, 525N Wolfe St, Baltimore, MD, 21205, United States, 1 443 514 7323; 2 Milken Institute School of Public Health, George Washington University, Washington, DC, United States; 3 School of Social Work, Howard University, Washington, DC, United States; 4 School of Pharmacy, University of Maryland, Baltimore, Baltimore, MD, United States

**Keywords:** digital health literacy, health behavior, cardiovascular disease, cardiovascular risk, vegetables, fruits, sugar-sweetened beverages, Black, African American, Latino, United States

## Abstract

**Background:**

Black and Hispanic adults in the United States face a disproportionately high burden of cardiovascular disease (CVD). Digital health literacy (DHL) may influence CVD prevention and management, yet its role in these populations is not well understood.

**Objective:**

This study aimed to examine associations between DHL and cardiovascular-related lifestyle behaviors, CVD, and CVD risk factors among Black and Hispanic adults.

**Methods:**

This was an exploratory analysis of survey data from a cross-sectional study among adults in Maryland; Virginia; and Washington, DC (March 2024-June 2024). DHL was measured using items from the Digital Health Literacy Instrument, and outcomes included self-reported CVD and risk factor diagnoses and lifestyle behaviors (physical activity and fruit-, vegetable-, and sugar-sweetened beverage intake). Multivariable regression models were used to assess associations, adjusting for sociodemographic and health literacy factors.

**Results:**

Among 1221 participants, the mean age was 44 (SD 16) years; most were female (n=766, 62.7%), insured (n=1065, 87.2%), and non-Hispanic (n=840, 68.8%) and identified as Black or African American (n=778, 63.7%). Higher DHL was associated with greater vegetable intake (incidence rate ratio 1.08, 95% CI 1.01‐1.15). Contrary to our hypothesis, DHL was positively associated with sugar-sweetened beverage consumption (incidence rate ratio 1.13, 95% CI 1.10‐1.25). DHL showed a significant nonlinear association with fruit intake (*P*=.01). No significant associations were observed with CVD or its risk factors.

**Conclusions:**

The relationship between DHL and cardiovascular-protective behaviors was mixed, suggesting that DHL may not be sufficient to promote consistent health-protective behaviors. The findings highlight the need for interventions that strengthen DHL while also addressing broader contextual and structural factors such as targeted digital marketing of unhealthy food and beverages, as well as environmental barriers. Longitudinal and experimental studies are needed to clarify causal pathways and inform equitable CVD prevention strategies.

## Introduction

Cardiovascular disease (CVD) remains the leading cause of death in the United States, with persistent racial and ethnic disparities in CVD risk, morbidity, and mortality [1]. Projections indicate that, between 2025 and 2060, the prevalence of CVD and its risk factors will rise among racially and ethnically minoritized populations, including Black and Latino adults, mainly due to higher baseline burden of risk factors [2]. Modifiable health behaviors such as healthy dietary patterns and regular physical activity are foundational to CVD prevention and management. However, the adoption of health-promoting behaviors remains suboptimal among adults in the United States and notably lower among non-Hispanic adults, adults with low educational levels, and those with low income [3,4].

Digital health literacy (DHL)—the ability to seek, understand, appraise, and apply health information from electronic sources to address or solve health-related problems [5]—has emerged as an increasingly important social determinant of health driven by the rise of telehealth and mobile health technologies [6]. However, individuals with limited digital access or DHL skills face significant barriers to benefiting from digital health resources [7,8]. These barriers can exacerbate preexisting gaps, particularly among high-risk groups such as Black and Latino adults [9]. Research has shown that DHL is essential in the adoption of health-promoting behaviors, including healthy diet and regular physical activity, which ultimately influence health outcomes [10-13]. Individuals with adequate DHL are more likely to use online health information to make informed health care decisions, communicate with health care providers through digital health platforms such as patient portals, and effectively use mobile health apps to manage their chronic conditions [14,15]. Conversely, limited DHL can impede timely health care access, reduce treatment adherence, and exacerbate existing health inequities [16].

Despite persistent racial and ethnic disparities in CVD and its risk factors and evidence that limited DHL poses barriers to digital health adoption [17,18], most research on DHL and health behavior has focused on youth and adolescent populations [15-17] or older adults [10,11,18]. This leaves a critical gap in understanding how DHL shapes health behaviors among working-age adults, particularly Black and Latino populations, who face a disproportionate burden of CVD. Moreover, research on DHL across ethnic groups is scarce, and findings on the role of DHL on cardiovascular health outcomes are mixed, underscoring the need for additional investigation.

To address this gap, the primary aim of this study was to examine how DHL influences CVD-related lifestyle behaviors, specifically physical activity and fruit, vegetable, and sugar-sweetened beverage (SSB) consumption, as well as CVD status, among Black and Latino adults living in Maryland; Virginia; and the Washington, DC, metropolitan area. This study is novel in focusing on mainly working-age adults in these communities and in assessing both dietary and physical activity behaviors alongside self-reported CVD outcomes, thereby extending previous research that largely focused on youth, older adults, or single behavioral domains. We hypothesized that higher DHL would be associated with high frequency of fruit and vegetable intake, more frequent physical activity, lower consumption of SSBs, and a reduced likelihood of CVD or CVD risk factor diagnosis.

## Methods

### Study Design and Setting

This exploratory cross-sectional study is a secondary analysis of data from the Community Engagement Alliance Against Disparities–Washington, D.C.; Maryland; and Virginia (CEAL-DMV). The CEAL-DMV study is an ongoing community-engaged research initiative led by the National Institutes of Health (NIH) focused on addressing cardiovascular health disparities and health behavior change among Black and Latino adults. The study protocol was obtained from the Johns Hopkins Institutional Review Board (IRB00338867). The analytic sample included all participants enrolled in the parent study (N=1221).

### Recruitment and Participant Selection

Participants were recruited from March 2024 to June 2024 through community partners, including local community-based organizations, health clinics, and outreach events. Convenience sampling was used to select participants from underserved Black and Latino neighborhoods, who are often underrepresented in research.

Eligible participants were adults aged ≥18 years who self-identified as Black, African American, or Latino and resided in Washington, DC; Maryland; or Virginia, as well as reporting proficiency in either English or Spanish. Individuals with cognitive impairments preventing informed consent or survey completion were excluded. Written informed consent was obtained from all participants (N=1221) before survey administration.

### Data Collection Procedures

Data were collected using interviewer-administered face-to-face surveys in participants’ preferred language (English or Spanish) at community events, health fairs, and partner organization sites. Trained interviewers followed standardized protocols emphasizing cultural competency, confidentiality, and accurate survey administration. Participants were asked their sociodemographic characteristics. Other variables assessed were DHL, health literacy, diagnosis of CVD and risk factors for CVD, and health behaviors including diet and exercise patterns. Survey items were programmed and administered via the REDCap (Research Electronic Data Capture; Vanderbilt University) software.

### Measures

Survey items were selected by the Community Engagement Technical Assistance Center (CETAC), the national coordinating and support hub for the NIH-funded Community Engagement Alliance (CEAL), an initiative aimed at addressing health disparities. CETAC supports CEAL teams nationwide by standardizing metrics and data collection through the Common Survey to assess impact and promote continuous improvement. Core domains on the Common Survey were demographics; social determinants of health; trust in health information; and post–COVID-19 condition, chronic disease, and mental health. The CEAL-DMV research team also included locally relevant items such as measures for DHL and trust in medical research.

### Sociodemographic Variables

Participants were asked their age, sex, gender, ethnicity, race, educational attainment, English-language proficiency, net household income, type of health insurance, and employment status, as well as the number of adults and children in their household. These variables were categorical and were analyzed accordingly.

### Health Literacy

The question “How well do you speak English?” was used to measure English-language proficiency, with response options including “like a native,” “very well,” “well,” “not well,” “not at all,” and “I don’t know.” Additionally, self-reported health literacy was measured using the Single-Item Literacy Screener. This tool asks, “How often do you need to have someone help you when you read instructions, pamphlets, or other written material from your doctor or pharmacy?” Responses are recorded on a 5-point Likert scale ranging from 1 (“never”) to 5 (“always”).

### DHL Instrument

DHL was assessed using 10 selected items from the Digital Health Literacy Instrument (DHLI), a validated instrument that operationalizes 7 skills—operational skills, navigation skills, information searching, evaluating reliability, determining relevance, adding self-generated content, and protecting privacy [19]. This study assessed participants’ competency across 3 dimensions of DHL—information searching, determining relevance, and evaluating reliability—using 10 items rated on a 4-point Likert scale (1=“very easy,” 2=“easy,” 3=“difficult,” and 4=“very difficult”). The responses were reverse coded (4=“very easy,” 3=“easy,” 2=“difficult,” and 1=“very difficult”). This included statements such as “When you search for information online about health topics, how easy or difficult is it for you to apply the information you found in your daily life?” and “When you search for information online about health topics, how easy or difficult is it for you to use the information you found to make decisions about your health?” While the original DHLI consists of 21 items, the parent study selected a subset to alleviate participant burden. In this sample, internal consistency reliability was excellent (Cronbach α=0.94).

### CVD and Risk Factor Status

The primary outcome was self-reported diagnoses of CVD and CVD risk factors. Participants were asked whether they had been diagnosed with a series of 33 chronic diseases and conditions, such as CVD, dementia, and hepatitis. These were presented in a list format, and participants were prompted to “select all that apply.” CVD status in our analysis was determined by evaluating participant self-reported diagnoses of cardiovascular risk factors (hypertension, high cholesterol, diabetes, obesity, sickle cell disease, sleep disorders, and substance abuse disorders) or CVD (coronary heart disease, heart failure, other heart or vascular disease, stroke, and thrombotic disorders). CVD status was coded as a binary variable: 1 for individuals reporting any CVD or CVD risk factor and 0 for those reporting none.

### Behavioral Outcomes

The secondary outcomes included participants’ patterns of physical activity and nutritional intake. Frequency of exercise was assessed using 1 question asking participants how many days per week they engaged in moderate to strenuous exercise, with responses ranging from 0 to 7 days. Participants who selected a range of 1 to 7 were then asked to indicate the approximate number of minutes of exercise per week. Nutritional intake was measured using 3 validated questions adapted from the National Health and Nutrition Examination Survey, which asked participants to recall how often they had consumed fruits and vegetables, as well as SSBs, in a typical week over the previous month. For each item, frequency was reported using the following categories: “never,” “1‐2 times per week,” “3‐4 times per week,” “5‐6 times per week,” “more than 6 times per week,” or “I don’t know” [20,21]. Standardized estimates were reported for behavioral outcomes focused on nutrition to better understand the effect size of each predictor.

### Bias

We minimized bias through the use of validated instruments (DHLI, Single-Item Literacy Screener, and National Health and Nutrition Examination Survey adapted items) and standardized survey measures developed by CETAC for the NIH CEAL initiative, the addition of locally relevant items to improve cultural appropriateness, the reduction of participant burden by limiting survey length, and reliability checks (Cronbach α=0.94 for DHLI items).

### Statistical Analysis

Descriptive analysis was used to examine participants’ sociodemographic characteristics. Means and SDs were calculated for continuous variables, and frequencies and percentages were calculated for categorical variables. There were 5 outcomes assessed: CVD or CVD risk factor, exercise patterns, patterns of eating vegetables, patterns of fruit consumption, and SSB intake. The distribution of the outcome variables was assessed using histograms and normality tests. CVD or CVD risk factor status was modeled as a binary variable, whereas exercise patterns, patterns of eating vegetables, fruit consumption patterns, and SSB intake were modeled as count data. Univariable logistic regressions were used to assess the relationships between the key predictor, DHL, and the outcome variable, CVD or CVD risk factor, as well as the relationship between other sociodemographic predictors and CVD or CVD risk factor.

To address the excessive zeros observed in respondents’ exercise patterns, we compared Poisson, negative binomial, and zero-inflated negative binomial models. Model diagnostics including the Vuong test, overdispersion test, and information criteria indicated underprediction of zeros by the Poisson and negative binomial models and a better fit of the zero-inflated negative binomial model. For patterns of eating vegetables and fruits and drinking SSBs, quasi-Poisson models were used to assess the bivariate relationships. Relationships found to be significant in the univariable analyses were included in the adjusted model for all outcomes using bidirectional stepwise variable selection. The adjusted models for patterns of consuming vegetables, fruits, and SSBs were modeled using quasi-Poisson models and generalized additive models allowing for nonlinear relationships between the predictors and the outcomes to be assessed. The variance inflation factor was used to check for multicollinearity excluding any variable with a factor of ≥5. Model evaluations and fit such as area under the curve for CVD or CVD risk factor, overdispersion checks, and residual assumptions were assessed. Multiple-comparison adjustment using the Bonferroni method was applied to control for type I error inflation across outcomes for the key independent predictor. We used complete case analysis as missingness was less than 5% on the independent and dependent variables of interest. The R software (version 4.3.0; R Foundation for Statistical Computing) was used for all analyses in this study.

### Key Independent Variable

The independent variable was DHL. Mean scores were calculated across all 10 items in the DHL measure to obtain a single score for each participant.

### Ethical Considerations

Ethics approval to conduct this research was obtained from the Johns Hopkins Medicine Institutional Review Board (IRB00338867). All study procedures were conducted in accordance with the ethical standards of the Johns Hopkins University ethics committee and all applicable institutional and national guidelines for human participant research [22]. Before data collection, informed consent was obtained from all participants. Each participant was provided with detailed information about the purpose of the study; the procedures involved; the potential risks and benefits; and their rights, including the right to withdraw at any time without negative repercussions. To ensure privacy and confidentiality, all participant data were deidentified and stored on secure, password-protected servers accessible only to authorized research personnel. No personally identifiable information was included in any reports. Each participant received a US $50 gift card for their time and engagement.

## Results

### Sociodemographic Characteristics

Table 1 summarizes the sociodemographic characteristics of the sample. The mean age of the participants was 44 (SD 16) years. Most participants identified as female (766/1221, 62.7%) and Black, African American, or African individuals (778/1221, 63.7%). American Indian, Asian, and Native Hawaiian individuals constituted 5.4% (66/1221) of all respondents. Participants of Hispanic ethnicity represented 31.2% (381/1221) of all respondents, and 49.5% (604/1221) of all participants reported that they did not speak English as well as native speakers. Almost half of all respondents had a high school education or lower (589/1221, 48.2%) and a household income of <US $35,000 (591/1221, 48.4%). Most participants had some form of health insurance coverage (1065/1221, 87.2%) and adequate health literacy (750/1221, 61.4%). Most of the participants (738/1221, 60.4%) had a part-time or full-time job, whereas 28.1% (343/1221) were unemployed. The average DHL score was 2.9 (SD 0.7) on a 4-point Likert scale.

**Table 1.** Sociodemographic characteristics (N=1221).

Characteristic	Values
Age (y), mean (SD)	44 (16)
Sex at birth, n (%)	
Female	766 (62.7)
Male	425 (34.8)
Missing	30 (2.5)
Ethnicity, n (%)	
Non-Hispanic	840 (68.8)
Hispanic	381 (31.2)
Race^a^, n (%)	
American Indian or Alaska Native	22 (1.8)
Asian	36 (2.9)
Black, African American, or African	778 (63.7)
Native Hawaiian or other Pacific Islander	8 (0.7)
White	130 (10.6)
Educational level, n (%)	
Lower than high school	146 (12)
High school or GED^b^	443 (36.3)
Some college or associate degree	311 (25.5)
Bachelor’s degree or higher	313 (25.6)
Missing	8 (0.7)
English-language proficiency, n (%)	
Like a native	604 (49.5)
Very well	374 (30.6)
Well	92 (7.5)
Not well	92 (7.5)
Not at all	37 (3)
Missing	22 (1.8)
Number of adult household members, n (%)	
1	385 (31.5)
2	378 (31)
3	204 (16.7)
≥4	144 (11.8)
Missing	110 (9)
Number of minor household members (children), n (%)	
0	245 (20.1)
1	229 (18.8)
2	169 (13.8)
3	81 (6.6)
≥4	33 (2.7)
Missing	464 (38)
Household income (US $), n (%)	
<15,000	314 (25.7)
15,000-34,999	277 (22.7)
35,000-74,999	349 (28.6)
≥75,000	264 (21.6)
Missing	17 (1.4)
Type of health insurance, n (%)	
No insurance	156 (12.8)
Medicaid	382 (31.3)
Medicare	152 (12.4)
Private health insurance	414 (33.9)
Other insurance plan	53 (4.3)
Multiple health insurance plans	64 (5.2)
Employment status, n (%)	
Part time	228 (18.7)
Full time	510 (41.8)
Unemployed	343 (28.1)
Other	119 (9.7)
Missing	21 (1.7)
Health literacy, n (%)	
Adequate	750 (61.4)
Inadequate	461 (37.8)
Missing	10 (0.8)
Digital health literacy (score of 1-4), mean (SD)	2.9 (0.7)

a Participants could select all that applied.

b GED: General Educational Development.

### Key Outcomes

Less than half (510/1221, 41.8%) of the participants had CVD or its risk factors. Most consumed vegetables (439/1221, 36%) and fruits (397/1221, 32.5%) 3 to 4 times a week. Among those who consumed SSBs, most (352/1221, 28.8%) consumed them 1 to 2 times a week, and most participants (296/1221, 24.2%) did not exercise weekly. These findings are summarized in Table 2.

**Table 2.** Distribution of outcome variables (N=1221).

Outcome	Participants, n (%)
CVD^a^ or CVD risk factor	
Yes	510 (41.8)
No	711 (58.2)
Vegetable intake	
Never	56 (4.6)
1‐2 times per wk	305 (25)
3‐4 times per wk	439 (36)
4‐5 times per wk	225 (18.4)
5‐6 times per wk	159 (13)
Unknown	37 (3)
Fruit intake	
Never	61 (5)
1‐2 times per wk	316 (25.9)
3‐4 times per wk	397 (32.5)
4‐5 times per wk	223 (18.3)
5‐6 times per wk	198 (16.2)
Unknown	26 (2.1)
Exercise (d per wk)	
0	296 (24.2)
1	88 (7.2)
2	146 (12)
3	223 (18.3)
4	148 (12.1)
5	137 (11.2)
6	49 (4)
7	125 (10.2)
Unknown	9 (0.7)
Sugar-sweetened beverage intake	
Never	269 (22)
1‐2 times per wk	352 (28.8)
3‐4 times per wk	260 (21.3)
4‐5 times per wk	158 (12.9)
5‐6 times per wk	148 (12.1)
Unknown	34 (2.8)

a CVD: cardiovascular disease.

### Associations Among DHL, CVD Status, and Behavioral Outcomes

In the unadjusted model, every unit increase in DHL was marginally associated with a 22% decrease in the odds of CVD and CVD risk (odds ratio 0.78, 95% CI 0.61-0.99). However, this relationship was attenuated after adjusting for age, sex at birth, race, ethnicity, educational level, and inadequate health literacy (odds ratio 0.97, 95% CI 0.73-0.128).

### Behavioral Outcomes

In the unadjusted models (Table 3), a unit increase in DHL was associated with an 8.4% increase in the expected frequency of eating vegetables (incidence rate ratio [IRR] 1.08, 95% CI 1.02‐1.15) and 9% increase in the expected frequency of eating fruits (IRR 1.09, 95% CI 1.03‐1.16). Similarly, a unit increase in DHL was associated with a 17% increase in frequency of SSB consumption (IRR 1.17, 95% CI 1.07‐1.28). However, an increase in DHL was not significantly associated with frequency of exercise per week.

After adjusting for age, sex at birth, race, ethnicity, level of education, and health literacy, DHL was significantly associated with increased frequency of consumption of vegetables (IRR 1.08, 95% CI 1.01‐1.15), fruits (IRR 1.09, 95% CI 1.02‐1.16), and SSBs (IRR 1.13, 95% CI 1.02‐1.25). Table 4 provides the associated IRRs and CIs.

**Table 3.** The unadjusted estimated effects of digital health literacy on the patterns of exercise and vegetable, fruit, and sugar-sweetened beverage consumption.

Outcome	IRR^a^ (95% CI)
Frequency of vegetable consumption	1.08 (1.02-1.15)
Frequency of fruit consumption	1.09 (1.03-1.16)
Exercise pattern	1.08 (1.00-1.17)
Frequency of sugar-sweetened beverage intake	1.17 (1.07-1.28)

a IRR: incidence rate ratio.

**Table 4.** The adjusted estimated effects of digital health literacy on the patterns of exercise and vegetable, fruit, and sugar-sweetened beverage consumption.

Outcome	IRR^a^ (95% CI)
Frequency of vegetable consumption	1.08 (1.01-1.15)
Frequency of fruit consumption	1.09 (1.02-1.16)
Exercise pattern	1.04 (0.95-1.14)
Frequency of sugar-sweetened beverage intake	1.13 (1.02-1.25)

a IRR: incidence rate ratio.

Additionally, DHL was found to have a nonlinear relationship with patterns of eating fruits in the adjusted model. The smooth term for DHL was significant (*F*_2.64_=3.99; *P*=.01). The effect of DHL on expected frequency of eating fruits was negative for DHL scores lower than 1.8, with the expected frequency of fruit intake falling below the average of 3 to 4 times per week. The effect was positive for DHL scores greater than 1.8, with expected fruit intake above the average, ranging from 5 to 6 times per week.

## Discussion

### Principal Findings

This study examined associations between DHL and CVD status, as well as engagement in CVD-related health behaviors, among a sample of Black and Hispanic adults in Maryland; Virginia; and Washington, DC. Prior work in these populations has linked DHL to the use of online health information [23]. Our findings partially supported the hypothesized relationships: higher DHL was associated with more frequent vegetable intake and higher consumption of SSBs, a behavior known to increase CVD risk. DHL had a nonlinear association with fruit intake and was not associated with exercise patterns, CVD, or its risk factors.

Digital tools and digital behavior change interventions have been shown to improve both digital literacy and dietary behaviors [24-27]. The relationship between DHL and fruit intake was nonlinear, with significant effects observed only at the lowest and highest ends of the DHL spectrum. This pattern suggests that the relationship between DHL and fruit consumption may be more pronounced among individuals with either very limited or very strong DHL skills. Evidence from previous studies supports the link between DHL and fruit or vegetable intake. For instance, a study among young American adults aged 18 to 20 years found that participation in a 24-week digital health intervention increased fruit intake compared to controls [27]. Similarly, a study on German adolescents reported that lower DHL was associated with higher odds of nondaily fruit and vegetable consumption [28]. It is possible that participants with higher DHL in this study sample had greater access to and ability to act upon online information about healthy eating, thereby facilitating improved dietary choices. These findings suggest that enhancing DHL may be an important pathway for promoting the adoption and maintenance of healthy lifestyle behaviors, including adequate fruit and vegetable intake.

Several potential mechanisms may help explain the counterintuitive association between DHL and greater SSB intake, although these remain speculative as they were not directly measured in this study. It is possible that individuals with higher DHL engage more frequently with online platforms, thereby increasing their exposure to targeted digital advertising. Previous research has shown that digital advertisements can shape health behaviors, particularly among racial and ethnic minority groups, through persuasive marketing strategies that influence beverage preferences and consumption [24,26]. SSB consumption is also shaped by broader structural and contextual influences. US-based research has shown that neighborhood characteristics [29], social norms [30], and policy interventions such as beverage taxes [31] can influence both attitudes and behaviors related to SSBs. These factors likely interact with but are not captured by measures of DHL alone. Future studies should explore how individual digital skills interface with commercial, environmental, and social determinants to better understand the complex drivers of dietary behavior.

DHL was not significantly associated with the presence of CVD or CVD risk factors or the frequency of weekly exercise. Although digital health technologies may support self-management of cardiovascular risk factors, evidence for their impact on outcomes remains mixed [5]. Previous research has demonstrated the beneficial role of DHL in promoting physical activity. For instance, a 2022 online survey of Turkish adults (N=404) found that DHL was significantly associated with higher physical activity levels [32]. Differences between this study’s US sample and the Turkish samples may explain these divergent findings. Our results also indicate that, while DHL may be associated with health behaviors such as diet, its relationship to disease outcomes remains unclear. Previous literature suggests that cardiovascular health is influenced by broader social realities such as economic instability, systemic discrimination, neighborhood conditions, and chronic stress, all of which extend beyond an individual’s ability to navigate digital health information [33].

The null findings may also reflect methodological considerations. First, CVD status was measured broadly as a composite of self-reported diagnoses of both risk factors (eg, hypertension, diabetes, and obesity) and conditions (eg, coronary heart disease and stroke), potentially masking more specific associations. Second, exercise was measured using a single-item question, which may not fully capture dimensions such as intensity, type, or context. Finally, unmeasured confounders (eg, health care access and neighborhood environment) and mediators (eg, motivation and self-efficacy) may have attenuated observed associations. These methodological constraints underscore the need for longitudinal research using more nuanced measures of both DHL and health outcomes to clarify the pathways linking digital health competencies to CVD outcomes and physical activity.

### Limitations

Our findings should be interpreted in light of some limitations. First, the cross-sectional design of this study limits the ability to establish temporality and, therefore, limits inferences about causal relationships between DHL and dietary or exercise patterns. Second, the sample was selected using convenience sampling and drawn exclusively from the Black and Hispanic communities in the Washington, DC; Maryland; and Virginia region. While this focus enabled examination of DHL in populations historically underrepresented in digital health research, it limits the generalizability of the findings beyond this geographic area and demographic context. Third, this study relied on self-reported single-item measures of fruit, vegetable, and SSB consumption; exercise; and CVD or CVD risk factors, which may introduce recall and social desirability bias. To mitigate these limitations, we adapted validated questions from national surveillance surveys and restricted recall to the previous 30 days to reduce memory burden. Detailed examples of foods and activities were provided, and participants were instructed to consider all settings (home, work, school, and restaurants) when responding. We also included an “I don’t know” option to minimize inaccurate responses and used categorical frequency measures to improve reliability. During analysis, we excluded uncertain and nonsubstantive responses (ie, “I prefer not to answer”). While residual bias may remain, particularly due to social desirability and recall bias, it is likely nondifferential and would attenuate rather than inflate the observed associations. While this approach reduced participant burden, it likely underestimated the multidimensional nature of these behaviors, including portion size, beverage type, and exercise intensity. Consequently, our findings related to these outcomes should be interpreted with caution, and future studies would benefit from using more comprehensive multi-item or objective measures. Fourth, although DHL scores demonstrated high internal consistency, this study used only a subset of items from the full DHLI. While this approach likely alleviated participant burden, it may have limited the ability to capture the full range of DHL skill domains, potentially overlooking more nuanced associations and limiting construct validity and the ability to explain unexpected findings. Despite these limitations, this study adds valuable insights into both the promise and pitfalls of DHL in relation to cardiovascular health behaviors.

### Conclusions

In line with previous literature, DHL was positively associated with some cardiovascular-protective dietary behaviors (fruit and vegetable intake). However, it was not associated with exercise frequency and a diagnosis of CVD or CVD risk factors and was unexpectedly linked to greater SSB intake. These findings suggest that, while DHL may support certain health-promoting behaviors, its relationship to health outcomes is complex and may be shaped by unmeasured contextual factors such as neighborhood environments and exposure to targeted marketing. Given the study’s cross-sectional design and limited geographic scope, as well as the use of a nonprobability sample of Black and Latino adults in the Washington, DC; Maryland; and Virginia region, these findings should not be generalized beyond this population.

To bridge this gap, interventions should not only enhance individuals’ DHL but also improve digital health environments by providing trustworthy, culturally relevant content; ensuring readability across literacy levels; incorporating multilingual and literacy-sensitive resources; and regulating exposure to commercialized or misleading health information. Future research should use longitudinal or experimental designs to assess causal pathways. Studies should also examine potential moderators such as trust in digital sources of information and social support. Additionally, interventions should improve the accessibility, reliability, inclusivity, and equity of digital health information environments. These steps may help reduce persistent health disparities and unintended consequences such as susceptibility to misleading online content and targeted marketing of unhealthy products.

## Abbreviations

CEAL: Community Engagement Alliance
CEAL-DMV: Community Engagement Alliance Against Disparities–Washington, D.C.; Maryland; and Virginia
CETAC: Community Engagement Technical Assistance Center
CVD: cardiovascular disease
DHL: digital health literacy
DHLI: Digital Health Literacy Instrument
IRR: incidence rate ratio
NIH: National Institutes of Health
REDCap: Research Electronic Data Capture
SSB: sugar-sweetened beverage
